# A better start literacy approach: effectiveness of Tier 1 and Tier 2 support within a response to teaching framework

**DOI:** 10.1007/s11145-022-10303-4

**Published:** 2022-06-12

**Authors:** Gail Gillon, Brigid McNeill, Amy Scott, Alison Arrow, Megan Gath, Angus Macfarlane

**Affiliations:** 1 Child Well-Being Research Institute, University of Canterbury, Private Bag 4800, Christchurch, 8140 New Zealand; 2 School of Teacher Education, Faculty of Education, University of Canterbury, Christchurch, New Zealand; 3 Better Start National Science Challenge, Liggins Institute, University of Auckland, Auckland, New Zealand

**Keywords:** Oral language, Phoneme awareness, Strengths-based, Early literacy, Teaching, Tier 2, Reading, Spelling

## Abstract

The Better Start Literacy Approach (BSLA) is a strengths-based approach to supporting children’s literacy learning in their first year of school. Previous research has shown the approach is effective at accelerating foundational literacy knowledge in children with lower levels of oral language. This study examined the impact of the BSLA for children with varied language profiles and across schools from diverse socioeconomic communities. Additionally, a controlled analysis of the impact of Tier 2 teaching within a response to teaching framework was undertaken. Participants included 402 five-year-old children from 14 schools in New Zealand. A randomised delayed treatment design was utilised to establish the effect of Tier 1 teaching. Analyses showed a significant Tier 1 intervention effect for phoneme awareness, letter-sound knowledge, non-word reading and non-word spelling. There was no difference in intervention effects across socioeconomic groupings. Children were identified for Tier 2 teaching after 10 weeks of Tier 1 implementation. The progress of 98 children in response to Tier 2 teaching was compared to 26 children who met Tier 2 criteria but received only Tier 1 teaching within this study. Children in the Tier 2 group scored significantly higher on phonological awareness, non-word reading, and spelling than the control group at the post-Tier 2 assessment point, after controlling for pre-Tier 2 scores. The results suggest that a proactive strengths-based approach to supporting foundational literacy learning in children’s first year of school benefits all learners. The findings have important implications for early provision of literacy learning support in order to reduce current inequities in literacy outcomes.

## Introduction

Children approach formal literacy instruction with wide variability in their foundational oral language skills that underpin reading and writing success. Factors related to children’s home linguistic and cultural environment, early childhood education experiences, cognitive abilities, and psychological and health status all influence their early literacy learning. Understanding reading and writing pedagogies that help reduce this variability through ensuring all children have strong foundational skills for literacy success is essential if we are to reduce current educational inequities (Mullis et al., 2017). Response to intervention (RTI) models (also termed multi-tiered systems of support (MTSS) models in the literature) are designed to improve the literacy outcomes of all learners and ensure that timely teaching support is provided. Although RTI models have been implemented widely in practice, there is marked variability across different models and in the fidelity of their usage within classroom settings. Further research is required to identify key elements of RTI approaches that enhance their efficacy and efficiency. For example, researchers have highlighted that a ‘wait to fail’ approach for children with dyslexia or other reading difficulties is still possible within some RTI models if movement between tiers of support is too slow or static (Catts & Hogan, 2020; Fuchs & Vaughn, 2012). There is also a need for more detailed analysis of the composition and impact of Tier 2 support and its connection with the classroom-wide curriculum (Wanzek et al., 2016).

The Better Start LiteracyAapproach (BSLA) is a comprehensive approach to advancing children’s phoneme awareness, orthographic knowledge, word decoding and encoding, vocabulary, and oral narrative skills in their first year of school. It is aligned with an RTI model, but we have adopted the term “response to teaching” framework since it is implemented from the outset of literacy instruction. The term more appropriately conveys how teachers monitor children’s response to the BSLA teaching and then scaffold, adapt activities, or increase teaching intensity as necessary to ensure all children progress towards their next steps for learning. Further, the concept of “intervention” is contrary to the more holistic perspectives of learning held by many indigenous communities (Toulouse, 2016) and the term may have negative associations for these groups where previous interventions have focused on alignment with the knowledge of the dominant culture. Evaluation of the BSLA response to teaching framework has shown that it is more effective in advancing early word reading and spelling development for children who entered English medium schools with lower levels of oral language than the teachers’ usual curriculum approach (Gillon et al., 2019, 2020). This current study extends these findings through a larger scale implementation of the BSLA across differing socioeconomic groupings, including children with both typical language development as well as those with lower-level language skills. In particular, the study evaluates the effectiveness of this response to teaching framework for younger and older children within the cohort to provide guidance regarding optimal implementation timing. The study also seeks to understand the additional benefits of providing Tier 2 (small group support) in addition to Tier 1 teaching (universal support) in children’s first year of schooling.

Within education and health communities, and particularly within the framework of supporting indigenous communities, there is a strong international movement towards taking a strengths-based perspective to supporting children with higher needs, their families, and their communities (Broski & Dunn, 2018; Cooper & Driedger, 2018; Savage et al., 2014; Schlechter et al., 2019). A strengths-based (or solution-based) perspective within an education context focuses on what children are achieving, the learning conditions, the teaching practices, and the family and community supports that lead to successful outcomes. The perspective positively recognises children’s efforts and their emerging capabilities and strengths (Fenton et al., 2015; Lopez & Louis, 2009). A strengths-based perspective does not imply that teachers simply identify children’s learning strengths and work on further increasing skills in these areas. Rather, it is a perspective that recognises children’s potential for learning, their resilience, and the benefits of positively engaging children’s families through building trust and partnerships (as opposed to a teacher or specialist expert hierarchical model of support; Toros & Falch-Eriksen, 2021).

Historically, we have viewed the wide variability observed in children’s foundational literacy skills when they enter school within a deficit or problem-based framework, focusing for example on the nature and severity of a child’s oral language problems or the limited nature of children’s alphabetic or print awareness skills from school entry assessments. However, framing assessments and teaching practices for children and their families within a strengths-based perspective has potential advantages (Climie & Henley, 2016; Fenton et al., 2015; Lee-James & Washington, 2018; Rappolt-Schlichtmann et al., 2018). The BSLA was specifically designed from a strengths-based perspective and aims to accelerate phoneme awareness, letter knowledge, word decoding and encoding skills, vocabulary, and oral narrative abilities for all children and to engage children’s families in positive ways from the outset of children’s literacy learning. This approach is consistent with a strengths-based philosophy of “empowering individuals to flourish” (Lopez & Louis, 2009, p. 2), since it is well established that these foundational oral language skills are critical to early reading and writing success, which in turn is predictive of later reading and educational achievement (Hjetland et al., 2017; Russell et al., 2018). RTI frameworks are aligned with strengths-based perspectives since they are characterised by high quality teaching, systematic monitoring of children’s progress, and increasing levels of teaching intensity to ensure learning success for all children. Within the BSLA response to teaching framework, we emphasise the importance of adapting teaching to suit learner needs in ways that celebrate children’s successful learning attempts while still identifying their next step for learning.

### Evidence for the BSLA

Gillon et al. (2019) found that the BSLA significantly accelerated the early literacy achievement of 143 children across seven classrooms with lower levels of oral language when compared to the usual literacy curriculum (where the usual literacy curriculum did include other types of structured phonic and phonological awareness programmes). Further analysis of the performance of all children (i.e., children with typical and lower levels of oral language) showed that children who had received the BSLA earlier in the school year had stronger reading and spelling than children who started the approach 10 weeks later (Gillon et al., 2020). The study reported here replicates and extends these studies with a new cohort of children, teachers, and families. This paper focuses on the children’s development of phoneme awareness skills and the transfer of these skills to reading and spelling in response to the BSLA teaching within the children’s first year at school. A child’s phonological awareness ability has consistently been shown to be a strong protective factor in supporting children’s early reading success (Wilson et al., 2021).

### Optimal timing of additional teaching support

The timing of when additional teaching support is provided for children who show a slower response to quality literacy instruction is important. There is good evidence that earlier support that addresses learners’ needs is best for enhancing reading outcomes. Wanzek and Vaughan’s (2007) meta-analysis showed that reading support addressing individual needs provided in kindergarten and first grade were more effective than reading interventions provided in second and third grades. Similarly, Lovett et al. (2017) showed that although the triple-focus reading intervention was effective in Grades 1 to 3, its efficacy was markedly stronger for children who received this type of support in earlier grades (particularly first grade). For many children, additional support is provided after a period of failure, such as towards the end of Grade 1 or even into children’s second or third grade at school when reading difficulties are obvious. In New Zealand, where this study was undertaken, the typical model for more intensive reading support is provided in children’s second year at school. However, if quality Tier 1 teaching is in place from school entry, and this includes the regular monitoring of children’s response to teaching of foundational literacy skills, such as phoneme awareness and early word decoding abilities, then teachers will be aware of children who require more support much earlier in their learning.

Given the critical nature of the timing of additional support, it is important to explore this issue within response to teaching frameworks. There is some evidence that allowing immediate access to higher tiers of support for the children with the highest need at initial assessment can enhance reading outcomes. Al Otaiba et al. (2014) compared two RTI models delivered in Grade 1. The traditional model identified children for Tier 2 support after eight weeks of Tier 1 teaching. The dynamic model identified children directly for Tier 2 based on their initial assessment results. The results showed that children supported through the dynamic model had significantly stronger reading skills at the end of the year. Other authors stress the importance of carefully monitoring a child’s response to quality Tier 1 instruction as a key component of providing timely support (Catts & Petscher, 2022; Miciak & Fletcher, 2020).

Another factor to consider is the impact of the age of children in their class or year group. Children who are young for their year group have, on average, lower scores than older peers in their year group on key foundational skills such as oral language (Norbury et al., 2016). Younger children within a year group are thus more likely to be identified for specialist education support, even when skills are developmentally appropriate (Gledhill et al., 2002). When assessment controls for age, there are benefits to starting school earlier (i.e., having exposure to more instructional time). Cornelissen and Dustman (2019) examined the cognitive (language and numeracy measures) and non-cognitive (physical, creative, behavioural) skills of 8000 children in their reception year in the United Kingdom. The researchers compared children of the same age but who had different school starting dates. Children who started school a month earlier exhibited increased scores at 5 and 7 years of age. The positive impact of an earlier school start date was more pronounced for boys from lower socioeconomic backgrounds.

Previous studies have not evaluated the impact of implementing an RTI approach on younger versus older children in the same age cohort. In New Zealand (where the current study is based), children typically start school on or around their 5th birthday. This study aims to compare the effectiveness of the BSLA for younger versus older children in their first year of formal reading instruction to gain a better understanding of the impact of age and time at school as variables influencing response to teaching approaches. Findings from the study will have important implications for improving the efficacy of the RTI and MTSS models.

### Tier 2 small group teaching

This study also seeks to extend our knowledge of the benefit of Tier 2 small group instruction that is aligned with Tier 1 teaching following a relatively short period (10 weeks) of quality Tier 1 instruction for 5-year-old children. In Truckenmiller and Brehmer’s (2021) review of Tier 2 interventions, the researchers noted that the most successful Tier 2 interventions were typically a full school year in length in order to adequately enhance children’s foundational learning skills for reading and writing success. Similarly, Wanzek and Vaughan’s (2007) meta-analysis was focused on interventions that included 100 or more sessions. The resources required to implement Tier 2 interventions at this intensity is substantial. Further research is required to examine more efficient models of providing this support. The provision of a framework that includes close alignment between Tier 1 and Tier 2 teaching in the first year of formal literacy instruction is a potential method for optimising the efficiency of Tier 2 teaching gains. Unfortunately, few studies have clearly documented the content of Tier 1 and Tier 2 teaching and how they are linked within the same RTI model (Wanzek et al., 2016), which limits our understanding of how to strengthen tiered teaching approaches. Other criticisms of research in this area that limit the generalizability of findings include insufficient detail regarding the number of teaching hours in each tier, the criteria used to identify children with Tier 2 support, and teaching fidelity across tiers (Wanzek et al., 2016). In addition to considering the timing of Tier 2 support and its alignment with Tier 1 teaching, it is important to document the specific types of small group phoneme awareness and word decoding strategies that lead to the greatest benefit for young learners. For example, Savage et al. (2018) directly compared two types of multicomponent teaching strategies within small group interventions for at-risk learners in Grade 1 (within the Canadian educational system). The teaching condition that resulted in sustained longer-term reading improvement incorporated the explicit linking of grapheme–phoneme patterns taught in isolation to connected text. The teachers in this condition also explicitly taught some of the complexities of the English language such as vowel digraph alternatives as opposed to the teaching of exception words as “sight words” through visual recognition strategies. Recent reviews have highlighted the variability in Tier 2 implementation (Arias-Gundín & Llamazares, 2021; Truckenmiller & Brehmer, 2021) and the need for continued research regarding specific details of effective Tier 2 reading instruction implemented in natural class teaching environments to guide teaching practice.

The following questions are addressed in this study:

1. Is the BSLA more effective in advancing children’s phoneme awareness and early reading and spelling skills than the usual literacy curriculum across a diverse range of school communities?
2. Does BSLA effectiveness differ based on child age within the year group at implementation?
3. What are the additional benefits of implementing the BSLA Tier 2 (small group) teaching for 5-year-old children with greater learning needs after 10 weeks of BSLA Tier 1 teaching?

## Method

A delayed treatment design was implemented. Following human ethics approval processes, meetings with school leadership teams, new entrants, and year 1 teachers were undertaken. Principals volunteered their schools’ involvement in the study. Fourteen schools from two cities in New Zealand (seven schools from Christchurch and seven in Auckland) were then randomly assigned for their teachers to receive Professional Learning and Development and in-class coaching support to implement BSLA first (Tier 1 followed by Tier 2; Group A schools, which commenced in August 2019) or to receive the approach second (Tier 1 followed by Tier 2; Group B Schools, commenced in February 2020). A new cohort of children entered into the study from Group A schools at the start of the new school year in February 2020.

Children in their first year at school, their class teachers, and their families (parent or other family member) participated in the project. Larger schools had multiple new entrants or year one classes within their schools participating in the project while smaller schools only had one class of children participating. Although all the children in these classes received the BSLA during their assigned research phase, data were only collected and analysed for the purposes of this study for children who had returned parental permission slips as per human ethics approval requirements. Data collected from all children with returned permission slips, including data collected for children with disabilities and children with English as a second or other language, were included in the analyses.

Parental consent was obtained for 411 children across the 14 schools. From this cohort, data across two assessment points (pre and post-10 weeks of BSLA Tier 1 teaching) were available for 402 children: 197 females (49.0%), and 205 males (51.0%). The mean age of this cohort at their first assessment was 63.8 months (SD = 3.4). There were 327 children in Group A and 75 children in Group B.

The ethnic composition of this cohort was 59.5% NZ European, 10.2% Māori, 8.2% Pasifika, 21.9% Asian, and 9.0% other ethnicities (note that children could affiliate with multiple ethnic groups). Of those with data available on languages spoken (n = 345), 15.9% were identified as speaking English as a second language and a further 7.8% were identified as speaking another language in addition to English. Baseline language skills were assessed using the Clinical Evaluation of Language Fundamentals Preschool (CELF-P2; Semel et al., 2006). A core language score was calculated that comprised the three subscales of expressive vocabulary, sentence structure, and word structure. The mean language score in this sample was 93.57 (SD = 17.68). The CELF-P2 core language standard score for 117 children (28.5% of cohort) was below 85, a threshold indicative of lower language skills.

**Fig. 1:**
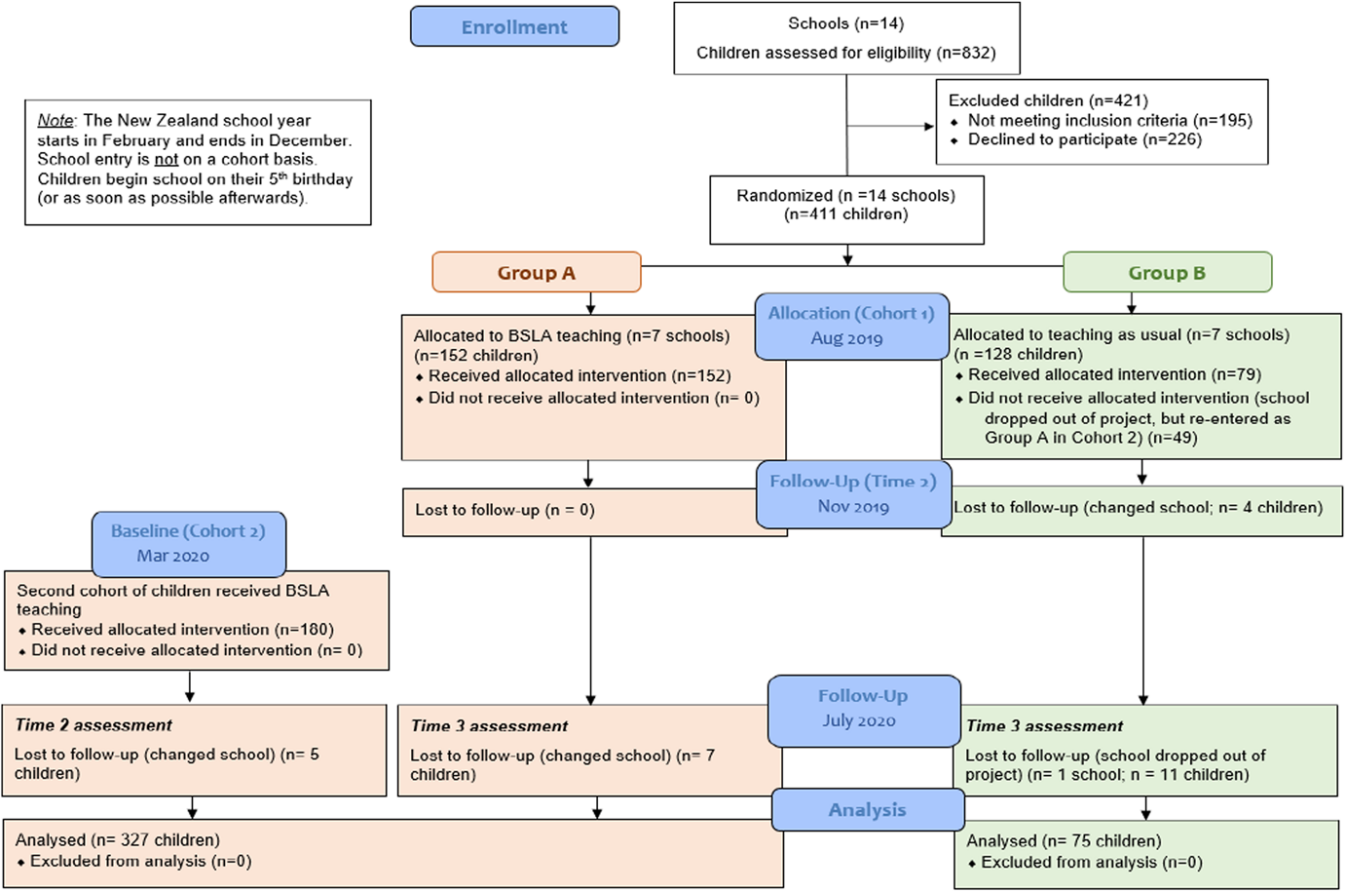
CONSORT flow diagram of participants

Of the children in the first intake of students in the study in August 2020 (n = 230), 212 children (92.2%) were retained to the third assessment point (post Group B receiving 10 weeks of BSLA Tier 1). The mean age of this group at first assessment was 64.5 months (SD = 3.0), noting that most children in New Zealand start formal schooling on their 5th birthday or within a couple of months of their 5th birthday depending on school entry policy. Attrition was a result of children changing schools and one school dropping out of the project. A CONSORT flow diagram is provided in Fig. 1. Fewer than 5% of cases had missing values on any given assessment task at each time point.

In New Zealand, each school is assigned a decile ranking from 1 to 10 that indicates the socioeconomic level of the community in which it is located (1 = lowest level). Schools in this study had been assigned a decile ranking of 2–10, with 50% of the schools being assigned a decile between 2 and 5 and the other 50% being assigned a decile between 6 and 10.

### Assessment measures

The research team developed novel online assessment tasks based on previous research trials (Gillon et al. 2019). Earlier versions of these online phoneme awareness tasks proved to be valid and reliable (Carson et al., 2015). The tasks were designed to:

- Describe children’s early progress in foundational oral language and early literacy skills to children’s families using strengths-based language;
- Help teachers to identify the next steps for learning for each child;
- Identify children who required more support after 10 weeks of teaching (i.e., required Tier 2 level support).

Gillon et al., (2019, 2020) provide further details of these online assessments. For the purposes of this study, the following assessments were implemented at baseline, following Group A receiving 10 weeks of the BSLA, and following Group B receiving 10 weeks of the BSLA. The tasks were also used to monitor children’s progress in response to Tier 2 level intervention, which commenced following the children’s participation in 10 weeks of Tier 1 BSLA. In addition, novel measures of children’s oral language abilities were used to monitor growth across the study period. As these were not experimental measures (i.e., we would not expect to see an impact on oral language in 10 weeks), the results of these assessments are not reported here, but the measures themselves and growth trends are described in detail in Gillon et al. (2022).

#### Phoneme awareness

The phoneme awareness tasks (Gillon et al., 2019) were presented via a touch screen iPad or laptop. An animated character (male voice with New Zealand accent) spoke to the children. The character asked children to complete various tasks and made general encouraging comments as the task progressed (e.g., “great work”, “you are trying hard”). The children were required to touch the screen (iPad) or the teacher/research assistant used a computer mouse to select the child’s response. The children’s responses were automatically recorded and a detailed response analysis for each child’s attempt was immediately available to the child’s teacher. The child’s response time for each item was recorded and the inbuilt time allowed 10 seconds for the child to respond prior to presenting the next item as the default option.

##### Phoneme identity

In this task, children were asked to select one of three pictures shown on the screen or iPad that started with the target sound. For example: “*Dog likes words that start with a /d/ sound. Can you help dog find words that start with his favourite sound. Which word starts with /d*/?” Pictures of a moon, a duck, and a whale are shown on the screen, and children must select the one that starts with /d/. Following two practice items, children completed 10 test items. Feedback was provided on the practice items only, indicating the correct response to each item. The phonemes tested were: /m/, /s/, /k/, /b/, and /f/ (two items tested each phoneme; see Appendix 1). Cronbach’s alpha, indicating the internal consistency across these 10 items, was 0.82. Test–rest reliability has previously been shown to be high for this task (Carson et al., 2015).

##### Phoneme blending

In this task, an animated character instructed the children: “*I am going to say one of these words very slowly. Click on the picture you think I am saying*.” For example, children were shown the images of a cake, a cape, and a ring, while listening to the character say the individual phonemes of /k/ /ei/ /k/. They were then required to click the image of the word they thought the character was saying. Following two practice items, children completed 12 test items (including, for example, mouse, lamp, and bun; all the items are provided in Appendix 1). After each practice item, the animated character indicated the correct response. Cronbach’s alpha for these 12 items was 0.83. Test–rest reliability has previously been shown to be high for this task (Carson et al., 2015).

##### Phoneme segmentation

In this task, children were asked to segment 3- and 4-phoneme words into individual phonemes. For example: children were shown an image of a dog and listened to the animated character sound out the phonemes /d/ /o/ /g/. They were then required to tap a box for each sound in the word. Following a practice item, children completed 12 test items (including, for example, tooth, cup, and soap; all items are provided in Appendix 1). After each practice item, the animated character indicated the correct response. Cronbach’s alpha for these 12 items was 0.57. Items with consonant blends and four phonemes proved too challenging for many children in this young age group. Test–rest reliability has previously been shown to be high for this task (Carson et al., 2015).

#### Letter sound knowledge

In this task, children listened to letter sounds and were asked to select the correct letter from six response options by tapping on the screen. Following two practice items, children completed 18 items assessing letter sound knowledge. Feedback was provided on the practice items only, indicating the correct response to each item. All the letters tested are provided in Appendix 1. Cronbach’s alpha for the 18 items was 0.89. The test–rest reliability has previously been shown to be high for this task (Carson et al., 2015).

#### Non-word reading

Children were asked to read 10 non-words and their responses were audio-recorded and scored. The non-words had one or two phonemes changed or deleted from a real word. Example items include tid, stap, and chom. Children received a point for every correct grapheme out of a total of 34 graphemes. Cronbach’s alpha across the ten items was 0.96. The task was administered by teachers or research assistants and scored by members of the research team. A second independent assessor scored a random selection of 20% of the non-word tasks at each assessment point. A one-way random ICC was used due to the first set of scores originating from multiple testers. The ICC for non-word reading (graphemes correct) ranged from 0.952 to 0.998 across assessment points, indicating high intertester reliability.

##### Non-word spelling

This task was not administered at baseline due to the young age of the children but was administered after 10 weeks of the BSLA for Groups A and B.

The children were asked to spell 10 non-words by writing each word on a record form that was read aloud to them. These items were the same non-words used for the reading task. Example items include mub, eps, and sib. Children received a score of 0, 1, or 2 for each grapheme. A score of 2 was given for a correct grapheme in the correct position, while scores of 1 were given for a correct letter that was in the wrong position, or for a b/d or p/q confusion. The non-word spelling task was out of a total of 68. Cronbach’s alpha across the ten items was 0.96. This task was administered by teachers or research assistants and scored by members of the research team. A second independent assessor scored a random selection of 20% of the non-word tasks at each assessment point. The ICC for non-word spelling (graphemes correct) ranged from 0.990 to 0.998 across assessment points.

### BSLA development and implementation

The BSLA implemented in the current study was based on a number of principles that can be aligned with a strengths-based perspective within a response to teaching framework. These include:

- Being proactive: A proactive approach ensures that children’s response to high quality literacy teaching is carefully monitored and that adjustments to teaching are made based on learners’ needs from their first year of schooling.
- Ensuring positive learning experiences: The self-teaching hypothesis for early reading (Share, 1995) highlights the cumulative power of children experiencing success in their early reading and spelling attempts. In the BSLA, teachers scaffold the task difficulty level to suit learner needs and provide positive explicit feedback on children’s attempts (e.g., reinforce which part of the word they successfully decoded) while identifying next steps for learning.
- Positive collaborations: Within the BSLA, teachers and literacy specialists work together to support literacy learning for all children. Speech–language pathologists (SLPs) are also frequently involved in supporting children with speech and language learning needs. Constructive and positive collaborations where each team member feels their area of expertise is valued help maximise the benefits of shared knowledge and experience to support children’s learning.
- Constructive reporting: BSLA teachers and literacy specialists are encouraged to carefully consider how assessment data are presented to parents. Presenting children’s data as percent correct (as opposed to percentage of errors), current level of achievement (as opposed to extent of delay), or number of phonemes spelt or read correctly in words (as opposed to word level error count) are examples of positive language use and shifts the focus to what the child is achieving. This prepares conversations on next steps required to enhance children’s reading and writing success in a positive way.
- Engaging parents: Parental engagement in reading stories with their children and teaching them early literacy skills (e.g., alphabet knowledge) is advantageous to their children’s early literacy learning (Hood et al., 2008). The BSLA therefore establishes contexts that positively facilitate family engagement, particularly for families that may find engagement within formal educational settings challenging. Developing trust and overcoming mistrust in the educational context; building positive relationships such as power-sharing by offering choices and flexibility in meeting times; resisting imposing a universal way of knowing; and using strengths-based, non-judgemental language in interactions are all examples of strengths-based perspectives for engaging parents (Gerlach et al., 2017) that are reinforced within BSLA.
- Culturally responsive: Respecting and valuing cultural differences and recognising the strengths of bilingual or multilingual development foster a strengths-based perspective to instructional practices. Gillon and Macfarlane (2017) provide examples of the types of cultural responsive practices in relation to phonological awareness and early literacy development that are used within the BSLA. The BSLA has also adopted the Hikairo Schema Primary, a culturally responsive teaching and learning framework (Ratima et al., 2020) that encourages teachers to teach in culturally responsive ways. The Hikairo Schema prioritises concepts from Māori culture (New Zealand’s indigenous people), which can benefit both Māori and non-Māori through creating inclusive learning environments. Three core elements are embedded in the schema (p15):
  1. Relevance. Learning that is aligned with children’s values and cultural and personal identities (e.g., including quality stories related to Māori culture and integrating Māori language and images into language games and activities) demonstrates relevance for children and their families;
  2. Balance of Power. Teachers help create learning environments that facilitate supportive relationships. For example, engaging children in open ended dialogue where they feel respected and confident enough to share their stories, ideas, or personal experiences related to reading texts;
  3. Scaffolding. Scaffolding techniques are used to help ensure each child experiences success in learning. For example, the teacher carefully scaffolds a literacy learning task so that the content is within the grasp of the learner using appropriate supports and resources as necessary for a successful learning outcome.
- Protective factors: Teachers implementing BSLA are encouraged to work with other educators, specialists, health professionals, and families to advocate for external influences of positive early literacy experiences. Internationally, there are common ecological and health factors that are associated with facilitating literacy learning success such as participation in quality early childhood education prior to school entry, access to quality children’s books, and ensuring the child is sleeping well and in good health (Mullis et al., 2017).

In adopting these principles of a strengths-based perspective on literacy teaching, the researchers co-constructed the implementation of effective instructional practices with class teachers to enhance children’s phoneme awareness, letter knowledge, word level reading, spelling, vocabulary, and oral narrative skills.

#### BSLA implementation support

School leaders actively encouraged and supported their teachers to implement the BSLA. The approach included quality online professional development for teachers. The research team worked with the children’s teachers through face to face workshops (where teachers were released from their class teaching to attend). Two literacy specialists (PhD qualified and trained in the BSLA by the lead researchers) were employed to work 60% of the hours of a full-time position each to provide coaching and mentoring support to the teachers throughout their implementation of Tier 1 BSLA. The amount of support each teacher received varied and was dictated by teachers’ confidence, skill, and demonstrated or requested need for support. High quality teaching resources, quality children’s story books, game activities, and lesson plans were provided to each teacher.

Workshops for the children’s parents and/or family members were also offered on multiple occasions to help meet the needs of working parents. Teachers and families were provided with decodable children’s texts. The research team wrote these texts, which were short stories that were culturally relevant to the New Zealand context and aligned with the BSLA class teaching activities. These readers had teaching notes at the back of each book. Families were encouraged to use these notes to help develop their child’s phoneme awareness, oral language, and vocabulary skills around the story theme.

##### BSLA Tier 1 universal teaching

Teachers implemented the BSLA with all children in their class using the BSLA materials provided. The BSLA teaching replaced their usual literacy programme. Structured 30-min lesson plans were provided for four days per week. These sessions were done at the whole class (large group) level with an average of 15 children. Differentiation within the lesson was achieved by teachers adjusting aspects such as task difficulty and scaffolding support based on children’s needs. The plans help to develop skills in three key areas:

1. Vocabulary learning through a carefully selected quality children’s storybook. Teachers used vocabulary elaboration teaching strategies where they provided a simple definition of a target word in the moment (adapted from Justice et al., 2005). Four to six interesting words per story were selected for elaboration and a definition was provided in the story book page on a sticky note to support the teacher.
2. Letter–sound (phonic) knowledge and phonological awareness teaching was integrated through games and explicit teaching activities for phoneme identity, phoneme blending, and phoneme segmentation. The activities selected were based on previous successful research trials (e.g., Carson et al., 2013; Gillon, 2000). Letters were introduced using larger clear font following a phonic scope and sequence that started with earlier developing speech sounds (e.g., m, d, p, and t) and consonant–vowel–consonant word patterns.
3. Ability to use emerging phoneme awareness and letter sound knowledge to decode and spell words was targeted through explicit teaching activities such as manipulating sound changes in words with grapheme tiles (e.g., Teacher: “If this word spells **mat,** show me **map.**” The child was required to change the last grapheme tile **t** in **mat** to the **p** grapheme tile and then to read the new word **map.** The teacher provided prompts as necessary to ensure the child was successful in the attempt). Reading target words in carrier phrases or simple sentences was also included in this activity segment, which was based on activities in the Gillon (2000) study.

In addition, a lesson plan for a 10–15 min daily small group reading session was provided using the BSLA decodable texts or similar texts available in the teachers’ classes. Multiple groups were run each day so that children received four small group reading sessions per week in Tier 1 teaching. This small group reading focused on helping children transfer their phoneme awareness and vocabulary knowledge from class activities to the reading process. The small group reading session was differentiated with children of similar needs grouped together based on assessment data and teacher judgement. There were a maximum of 5 children per group.

##### BSLA Tier 2 small group focused teaching

Following 10 weeks of BSLA teaching in Tier 1, children were identified for Tier 2 support. Previous research examining selection methods for Tier 2 reading intervention concluded that a case-by-case approach using children’s individual data from a range of measures (as opposed to pre-determined benchmarks or growth rate models) for Tier 2 selection is recommended, particularly for English language learners (Richards-Tutor et al., 2013). A case-by-case basis for Tier 2 selection was therefore adopted in the current study. Identification included inspection of children’s post-Tier 1 phoneme awareness, letter knowledge, and non-word reading and spelling performance. In particular, children who showed evidence of struggling to transfer emerging phoneme awareness and letter knowledge to reading or spelling attempts (low non-word reading and spelling performance) were identified. Low non-word reading and spelling performance was defined as not consistently using or representing the first phoneme in non-word reading and spelling attempts. Teachers’ observations of these children’s progress during class Tier 1 BSLA activities confirmed that these children would benefit from Tier 2 support.

The BSLA Tier 2 support involved 30-min small group instruction (up to 4 children) four times a week. A literacy specialist or SLP (trained by the research team) or a research team member implemented Tier 2 in a classroom setting or in a ‘breakout’ space (a number of the classes were configured in open plan or flexible learning environments). Tier 2 was typically implemented in a morning teaching session during a time when all the children in the class were involved in some type of small group language activity. These lessons focused on (a) enhancing children’s phoneme segmentation, blending, and phoneme manipulation skills and (b) the transfer of phoneme awareness and orthographic knowledge to the ability to read and spell words in both isolation and in connected text. The activities were directly aligned with the types of phoneme awareness and small group reading activities that children had received in Tier 1, but the teaching in Tier 2 was more intensive and targeted towards children’s specific learning needs. These children continued to participate in other Tier 1 BSLA teaching activities such as shared book reading. Teaching intensity within Tier 2 support was enhanced by ensuring close alignment between Tier 1 and Tier 2 teaching content (i.e., increasing exposure) and the small group context (i.e., increased opportunity for individualised scaffolding).

### Teaching fidelity

#### Tier 1

Teachers were asked to complete a fidelity checklist after each lesson, indicating the activities that were included in their class/large group session. They were also asked to check whether they considered their children showed high engagement, average/variable engagement, or low engagement in the day’s group lesson. From week 3–10 in the BSLA Tier 1 teaching, teachers were also asked to audio record one lesson per week for the phoneme awareness activities in the class session. Ten percent of the 126 recordings received were randomly selected and reviewed by an independent assessor. An assessor who was familiar with the key components of BSLA was trained by a member of the research team to complete fidelity checklists for the randomly selected recorded lessons. Each intervention component (listed in Table 1) was recorded as present or absent by the independent assessor.

**Table 1:** Percentage of lessons that incorporated key BSLA teaching elements as reported by teachers’ checklists or as independently verified through listening to audio recordings of teaching activities

Class/large group key teaching components	Percent of lessons including component	
	Checklist data (n = 567)	Audio data (n = 14)
• Reading/summary of the week’s storybook and vocabulary elaboration techniques	87%	NR
• At least one skill building activity that targeted phoneme identification	91%	100%
• At least one skill building activity that targeted phoneme blending and segmentation	90%	100%
• At least one activity that targeted phoneme manipulation with graphemes	86%	93%
• Small group reading activity implemented	86%	NR

Small group reading activity Tier 1 key components	Checklist data (n = 448)	
• Included explicit instruction of new phonic patterns introduced in the reader	76%	NR
• Used decodable text suitable to the child’s skill level	84%	NR
• Practiced reading and spelling target words with grapheme tiles before reading connected text	72%	NR
• Included teaching of high-frequency words	68%	NR

Tier 2 BSLA teaching key components	Checklist data (n = 410)	Audio data (n = 19)
• A phoneme segmentation/blending activity with at least six target words	99%	90%
• A phoneme manipulation activity with grapheme tiles and at least six phoneme/grapheme changes	99%	95%
• A small group reading activity using a phonological decoding strategy to decode phonetically regular target words in connected text	96%	95%

*NR* not recorded

Teachers reported that children’s engagement with the Tier 1 activities was high for 60% of the lessons, average or variable for 38% of lessons, and low for 2% of lessons. Teachers reported that the total instructional time for class/large group sessions was 30 min or more in 77.3% of lessons. The selection of audio recordings had a mean length of 24 min (range 15–35 min) for the class/large group session. Variability was evident as some teachers selected to engage children in the storybook and vocabulary extension activities at different times of the day, allowing for more time on the phoneme awareness activities during their literacy teaching time.

#### Tier 2

After each lesson, Tier 2 instructors completed a fidelity checklist on student engagement and the teaching activities implemented. Instructors were also asked to audio record one lesson per week. Twenty percent of the 89 recordings received were randomly selected for review by an independent assessor who was trained in the key components of Tier 2 teaching. The assessor recorded each intervention component as present or absent (see Table 1).

The instructors reported that student engagement was high for 87% of lessons and average or variable for 13%. The mean length of instructional time reported by instructors was 32 min (range 20–40 min). The selected audio recordings had a mean length of 29.66 min for the Tier 2 small group sessions (range 19.39–38.14 min).

### Usual teaching curriculum

The teachers implemented the New Zealand English curriculum. The Ministry of Education provides Literacy Learning Progressions regarding expected children’s progress to guide teachers. Free teaching resources such as children’s readers are provided to all state schools. However, teachers can choose how the curriculum is implemented and the types of children’s readers or phonological awareness programmes that they select to use. Shared book reading, language-rich play contexts, and small group reading instruction are common teaching activities within the usual class language curriculum. Teachers from Group B reported that their usual literacy curriculum did include some type of structured phonics and phonological awareness programme. This is consistent with recent survey data involving teachers in New Zealand Year 0–3 classes. Chapman et al. (2018) found that 90% of junior school teachers reported using a phonics type programme and 77% of these teachers reported using “explicit instruction” strategies to develop children’s phonics (letter–sound) knowledge.

### COVID 19 disruptions

The first phase of the project (August to November 2019) was implemented in usual teaching environments. However, the second phase, which began in early February 2020, after the long summer break in New Zealand, was interrupted by the COVID 19 pandemic. All schools in New Zealand closed for at least six weeks between late March 2020 and May 2020. During this time, teachers engaged with their new entrant and year 1 children online and through home-based learning activities. This affected the originally planned research design in three main ways.

1. Participant numbers: One large school that was assigned to receive the BSLA second (Group B school), and was only three weeks into implementing the BSLA when the COVID-19 lockdown commenced, withdrew from the study. Teachers in Group A, who had already implemented the BSLA (between August and November 2020), implemented the BSLA with a new intake of children into the study who commenced school in February 2020. They continued to implement some strategies from the BSLA online with their children during the lockdown period and when they returned to school. This led to a large difference in participant numbers between Group A and Group B in the analyses.
2. Changes to planned assessment schedule: The COVID-19 lockdown period led to a change in the planned assessments. We implemented a further pre-Tier 2 assessment point to ensure we had recent assessment data to evaluate any observed changes in children’s progress when additional support was implemented.
3. Additional support for Group B: We provided additional research assistant support for some of the teachers in Group B who remained in the study after the lockdown period to help these teachers re-engage with the BSLA with their children.

## Results

### Group comparisons at baseline

Preliminary analyses compared Group A and Group B on baseline characteristics to determine the equivalence between the children in each group. One-way analyses of variance indicated that there were no significant differences between Groups A and B in terms of CELF-P language scores (*F*(1405) = 1.74, *p* = 0.19; Hedges’ *g* = 0.17) or school decile (*F*(1408) = 0.54, *p* = 0.46; Hedges’ *g* = 0.09). The groups did differ in terms of age, with the children in Group B (M = 64.62, SD = 4.44) being one month older on average than the children in Group A (M = 63.62, SD = 3.10) (*F*(1407) = 5.44, *p* = 0.02; Hedges’ *g* = 0.29). There was a similar proportion of children who spoke English as a second language across groups (Group A: 16.1%; Group B: 15.4%; χ^2^(1) = 0.02, *p* = 0.53; φ =  − 0.007). In the full sample of children with data available at Time 1 and Time 2, Group A and B did not differ on any of the assessment tasks at baseline (means and standard deviations are provided in Table 2). Baseline scores for all tasks have been included as covariates in our statistical models.

**Table 2:** Means and standard deviations by group and assessment point

	Time 1 Baseline Pre-BSLA Mean (SD)				Time 2 Post-Group A receiving BSLA for 10 weeks Mean (SD)					Time 3 Post-Group B receiving BSLA for 10 weeks Mean (SD)		
	Group A Cohort 1 (n = 152)	Group A Cohort 2 (n = 175)	Group B (n = 75)	Hedges’ *g* (A vs. B)	Group A Cohort 1 (n = 152)	Group A Cohort 2 (n = 175)	Group B (n = 75)	Hedges’ *g* (A vs. B)		Group A Cohort 1 (n = 145)	Group B (n = 67)	Hedges’ *g* (A vs. B)
Phoneme awareness	17.28 (5.99)	16.25 (6.80)	16.05 (5.39)	0.10	23.29 (4.91)	23.75 (6.20)	20.01 (6.31)	0.61		25.26 (5.13)	24.75 (4.83)	0.10
Letter sound knowledge	12.73 (4.56)	10.62 (5.28)	12.13 (4.84)	0.11	15.43 (3.04)	15.34 (3.49)	14.32 (4.02)	0.31		15.94 (2.82)	16.24 (2.03)	0.12
Non-word Reading	5.83 (8.82)	4.25 (8.08)	3.56 (6.69)	0.18	16.66 (10.50)	16.63 (11.49)	10.66 (9.76)	0.55		21.39 (11.88)	18.61 (10.15)	0.24
Non-Word Spelling	–	–	–	–	40.68 (18.82)	39.51 (20.88)	29.43 (19.16)	0.53		46.81 (19.71)	43.38 (18.31)	0.18

*BSLA* Better Start Literacy Approach

### Overview of statistical analyses

To investigate the differences in growth over time between Group A and Group B, we used hierarchical linear models (HLM) to account for the clustered nature of the data (Hox, 2019; Raudenbush & Bryk, 2002). We examined student assessment data nested within classrooms, which were nested within schools. Our final HLM models were built following the standard sequential process that starts with an unconditional model and builds progressively towards a final model. Our unconditional models for each dependent variable (phonological awareness, letter sound knowledge, non-word reading, and non-word spelling) indicated variance at both the classroom and school levels beyond variance attributable at the student-level (intraclass correlation coefficients: classroom = 0.003–0.16, school = 0.02–0.11), indicating that HLM was appropriate. The conditional (predictor) hierarchical models examined whether post-test student-level performance (Time 2 or Time 3) was explained by the predictors of group (A vs. B), age, or the interaction between group and age, with the covariates being school decile and pre-test scores (e.g., Time 1). Interaction terms were excluded from the final models when they were not significant. We expected to see significant differences between groups at Time 2, when Group A had received BSLA teaching and Group B had not. We anticipated that at Time 3, once Group B had also received BSLA teaching, we would no longer find significant differences in assessment scores between the groups.

Within the context of most children starting school on their 5th birthday or within a couple of months after their 5th birthday, children were grouped into two categories based on age—those aged 5y3m and below (50.9% of the sample) and those aged 5y4m and above (49.1%). Hierarchical models were run using PROC MIXED in SAS.

### Phonological awareness

A composite measure of phonological awareness was created by combining scores on phoneme identity, phoneme blending, and phoneme segmentation, as these were found through a principal components factor analysis to load onto a single factor. This factor had an eigenvalue of 1.80 and accounted for 60% of the variance in scores (factor loadings ranged from 0.70 to 0.82 at the first assessment point).

Our hierarchical model predicting phonological awareness at Time 2 (after Group A had received BSLA teaching) indicated a significant effect of Group (*F*(1345) = 18.39, *p* < 0.001), as well as a significant Group*Age interaction (*F*(1345) = 7.17,* p* = 0.008). Group A scored significantly higher than Group B at Time 2 (see Table 2); however, this difference between groups was only significant for the younger age group (*p* < 0.001) and not for the older age group (*p* = 0.19). The HLM coefficient estimates and standard errors are presented in Table 3.

**Table 3:** HLM results for post-test assessment scores

Assessment task	Assessment point	Parameter	Coefficient estimate	Standard error	Log likelihood ratio test^a^
Phonological awareness	Time 2	Group	1.14	0.86	χ^2^(1) = 28.5, *p* < 0.001
		Age	− 1.48	1.15	
		Group*Age	3.38	1.26**	
	Time 3	Group	0.28	0.65	χ^2^(1) = 2.9, *p* = 0.09
		Age	0.58	0.63	
Letter sound knowledge	Time 2	Group	1.16	0.43**	χ^2^(1) = 31.7, *p* < 0.001
		Age	1.46	0.28***	
	Time 3	Group	− 0.43	0.46	χ^2^(1) = 1.9, *p* = 0.17
		Age	0.38	0.33	
Non-word reading	Time 2	Group	4.01	1.52**	χ^2^(1) = 11.3, *p* < 0.001
		Age	0.87	0.94	
	Time 3	Group	0.51	1.57	χ^2^(1) = 6.8, *p* = 0.009
		Age	1.69	1.51	
Non-word spelling	Time 2	Group	8.91	3.35**	χ^2^(1) = 14.3, *p* < 0.001
		Age	− 3.12	1.98	
	Time 3	Group	4.31	2.84	χ^2^(1) = 20.1, *p* < 0.001
		Age	− 4.08	2.68	

All models control for school decile and pre-test scores

* *p* < 0.05; ** *p* < 0.01; *** *p* < 0.001

^a^Model fit is presented for each final model as a log likelihood ratio test comparing the final model against the model containing only covariates (school decile and Time 1 scores)

**Fig. 2:**
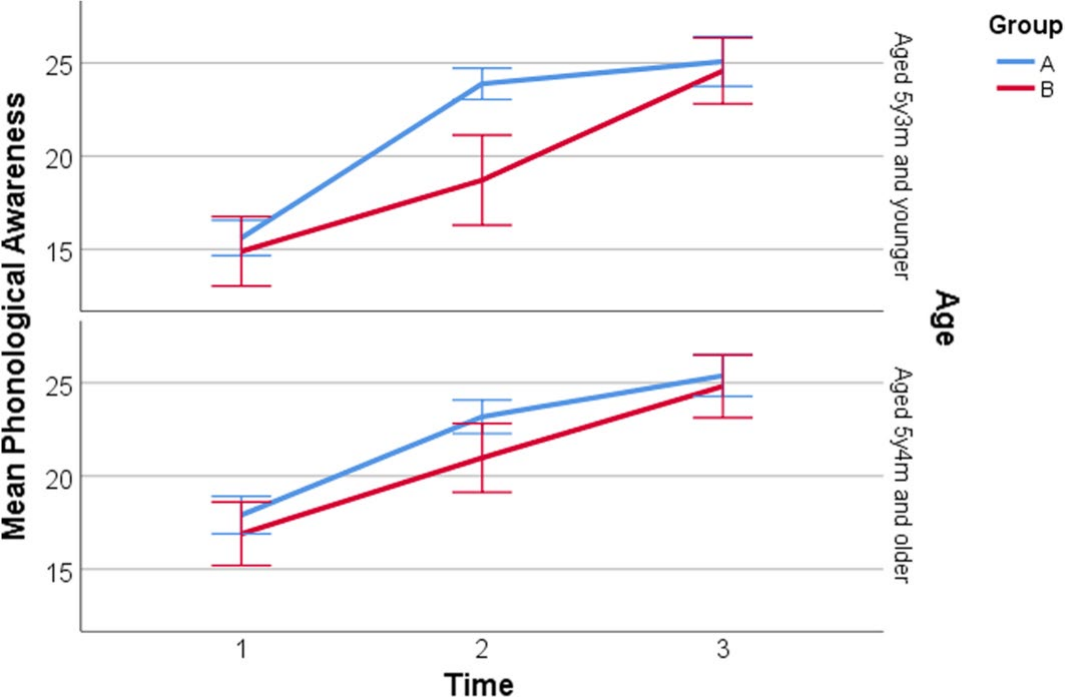
Phonological awareness by age and group over time

The model predicting phonological awareness at Time 3 (after Group B had received BSLA teaching) indicated no significant differences by Group or by Age, suggesting that once both groups had received BSLA teaching there were no significances differences between groups in phonological awareness. Mean phonological awareness scores by group and age over time are displayed in Fig. 2.

**Fig. 3:**
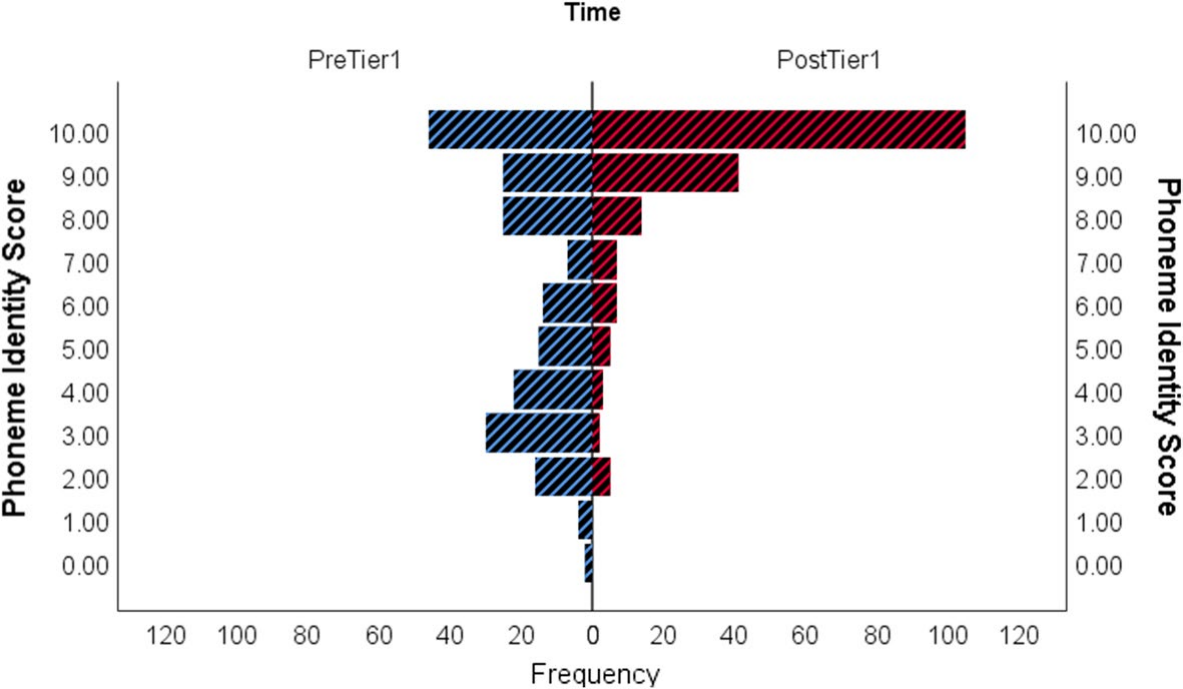
Initial phoneme identity scores pre-Tier 1 and post-Tier 1 for children aged 5 years 3 months and younger

**Fig. 4:**
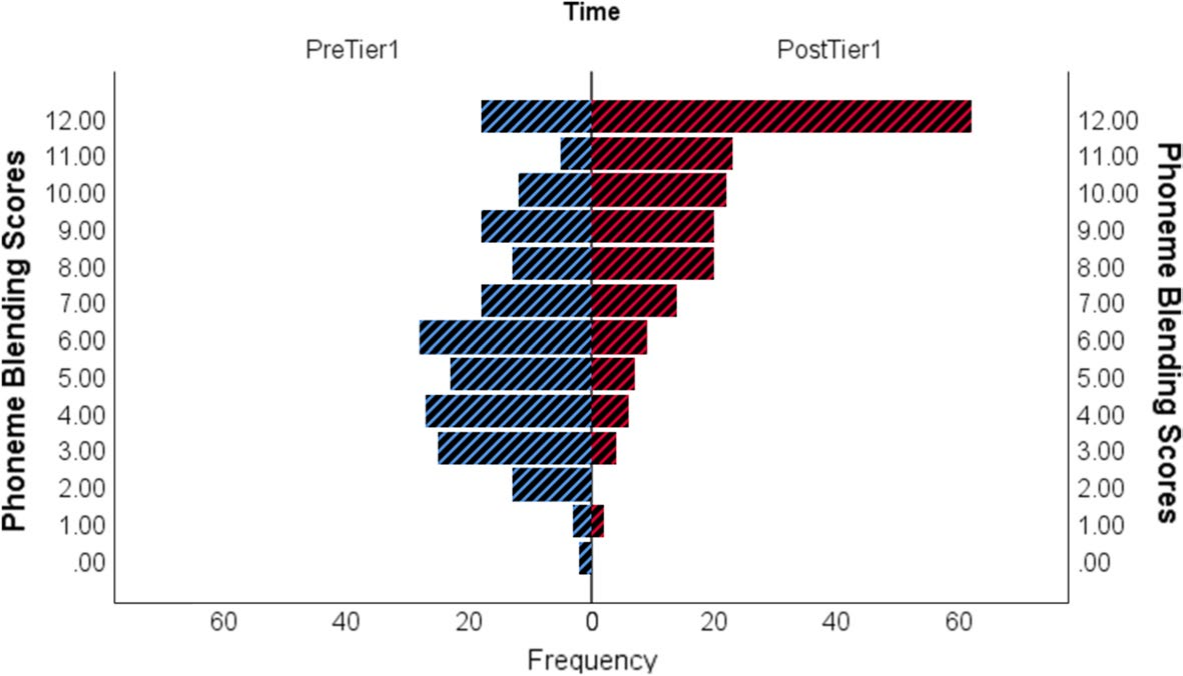
Phoneme blending scores pre-Tier 1 and post-Tier 1 for children aged 5 years 3 months and younger

Figure 3 shows the distribution of initial phoneme identity scores for all children aged 5y3m and younger at their pre-Tier 1 assessment (prior to receiving the BSLA) and their distribution of scores post-Tier 1 (after receiving the BSLA). Figure 4 shows similar distributions for phoneme blending scores. These figures demonstrate the large shift in the distribution of scores between pre- and post-BSLA teaching.

### Letter sound knowledge

Our hierarchical model predicting letter sound knowledge at Time 2 indicated a significant effect of Group (*F*(1345) = 7.19, *p* = 0.008) and Age (*F*(1345) = 27.79, *p* < 0.001). As there was no Group*Age interaction, this term was excluded from the final model. Group A (who had received BSLA teaching) scored higher in letter sound knowledge than Group B (who had received classroom teaching as usual) and younger children scored higher than older children. The HLM results are provided in Table 3. The model predicting letter sound knowledge at Time 3 (once Group B had received BSLA teaching) showed no significant differences by group in letter sound knowledge (*F*(1178) = 0.89, *p* = 0.35). There were also no differences by age interactions with age (see Table 3).

The finding of younger children scoring higher than older children at Time 2 was unexpected, and as a result we conducted further analysis in order to understand this outcome. Although the interaction of Group*Age was not significant (*F*(1346) = 0.51, *p* = 0.48), comparisons of least squares mean differences indicated that the children in Group A aged 5.3 and younger scored significantly higher than the older children in Group A, as well as both Group B age groups (*p*’s < 0.02). Thus, this main effect appears to be largely driven by the performance of younger children in Group A, while the two Group B age groups did not differ significantly from one another (*p* = 0.13).

### Non-word reading

The model predicting non-word reading at Time 2 showed a significant effect of Group (*F*(1340) = 7.00, *p* = 0.009) and no differences by Age (*F*(1340) = 0.86, *p* = 0.35). Group A scored significantly higher on non-word reading at Time 2 than Group B (see Table 2). At Time 3, once Group B had received BSLA teaching, there were no significant differences between groups in terms of non-word reading scores (*F*(1177) = 0.11, *p* = 0.74). The HLM results are provided in Table 3. There were no group by age interactions at either assessment point and these terms were not included in the final models.

### Non-word spelling

Non-word spelling was not assessed at Time 1 due to the children’s young age. We therefore were unable to control for Time 1 spelling scores in the prediction of these models. Our hierarchical model predicting spelling scores at Time 2 indicated a significant effect of Group (*F*(1340) = 7.00, *p* = 0.009). Group A, who had received BSLA teaching, scored significantly higher on spelling than Group B (see Table 2). Once Group B had received BSLA teaching at Time 3, there were no longer significant differences between groups (*F*(1181) = 2.30, *p* = 0.13). There were no differences by age or interactions with age at either time point (see Table 3).

### Tier 2

A total of 104 students (25.4% of cohort) were selected to participate in BSLA Tier 2. Data was available pre- and post-Tier 2 for 98 of these children. This group of children had a mean CELF language score of 87.9 (SD = 16.64) and 15.4% spoke English as a second language. When excluding children who spoke English as a second language, 31.2% of Tier 2 children met the threshold for low language abilities in English (scores below a standardised score of 85 on the CELF-P). A group of children who entered the study in the second cohort of the project (in 2020) were selected as a Tier 2 control group based on their post-Tier 1 results (n = 26). These children received Tier 1 BSLA but did not receive Tier 2 support during the study.^1^ These children received classroom teaching as usual instead of Tier 2 support. This control group of children had a mean CELF language score of 91.4 (SD = 14.5) and 15.8% spoke English as a second language. The Tier 2 group and the control group did not differ significantly in terms of baseline CELF scores (*F*(1127) = 0.93, *p* = 0.34; Hedges’ *g* = 0.21).

Both the children who participated in Tier 2 small group support and those in the control group completed a follow-up assessment a mean of 17 weeks (SD = 3.6 for Tier 2 and 8.2 for controls) after their Time 2 assessment in the study. Table 4 provides the means and standard deviations pre- and post-Tier 2 for children in Tier 2 and the control group on phonological awareness, letter–sound knowledge, and non-word reading and spelling. The two groups did not differ significantly on any of these assessments pre-Tier 2.

**Table 4:** Means and standard deviations for pre- and post-Tier 2 assessment tasks

	Pre-Tier 2 Mean (SD)			Post-Tier 2 Mean (SD)		
	Tier 2 (n = 98)	Control (n = 26)	Hedges’ *g*	Tier 2 (n = 98)	Control (n = 26)	Hedges’ *g*
Phonological Awareness (/34)	20.50 (5.64)	19.62 (7.53)	0.14	24.92 (4.78)	22.04 (5.32)	0.59
Letter–sound (/18)	14.08 (3.71)	12.85 (4.76)	0.31	15.69 (2.49)	14.26 (3.82)	0.51
Non-word Reading (/34)	8.95 (8.07)	7.38 (8.93)	0.19	20.7 (9.05)	12.08 (11.59)	0.90
Non-word Spelling (/68)	26.48 (16.76)	24.15 (21.90)	0.13	47.96 (14.81)	(21.35)	0.77

Tier 2: Teaching comprised BSLA focused small group support in addition to BSLA Tier 1 teaching activities. Control: teaching comprised BSLA Tier 1 teaching activities only

To examine the differences between the groups at the post-Tier 2 assessment point, we again used HLM; however, due to an insufficient number of children per class to cluster at this level, we modelled student-level assessment data nested within schools. ICCs for school-level variance ranged from 0.15 to 0.26 across tasks. In our conditional hierarchical models, we controlled for pre-Tier 2 scores and school decile and included Tier (Tier 2, control) as a predictor.

The hierarchical models predicting phonological awareness, non-word reading, and spelling all indicated a significant effect of Tier 2 (phonological awareness: *F*(1106) = 5.14, *p* = 0.03; non-word reading: *F*(1109) = 24.67, *p* < 0.001; spelling: *F*(1110) = 15.97, *p* < 0.001). The model predicting letter–sound knowledge indicated no significant effect of Tier 2 (*F*(1106) = 1.45, *p* = 0.23). HLM results are provided in Table 5. Children in the Tier 2 group scored significantly higher on phonological awareness and non-word reading and spelling than the control group at the post-Tier 2 assessment point, after controlling for pre-Tier 2 scores.

**Table 5:** HLM results for effect of Tier (Tier vs. control) on post-Tier 2 assessment data

Assessment task	Coefficient estimate	Standard error	Log likelihood ratio test^a^
Phonological awareness	1.81	0.80*	χ^2^(1) = 6.4, *p* = 0.01
Letter–sound knowledge	0.58	0.48	χ^2^(1) = 1.8, *p* = 0.18
Non-word reading	7.40	1.49***	χ^2^(1) = 25.2, *p* < 0.001
Non-word spelling	10.44	2.61***	χ^2^(1) = 18.8, *p* < 0.001

*p < 0.05; ***p < 0.001

^a^Model fit is presented for each final model as a log likelihood ratio test comparing the final model against the model containing only covariates (school decile and Time 1 scores)

**Fig. 5:**
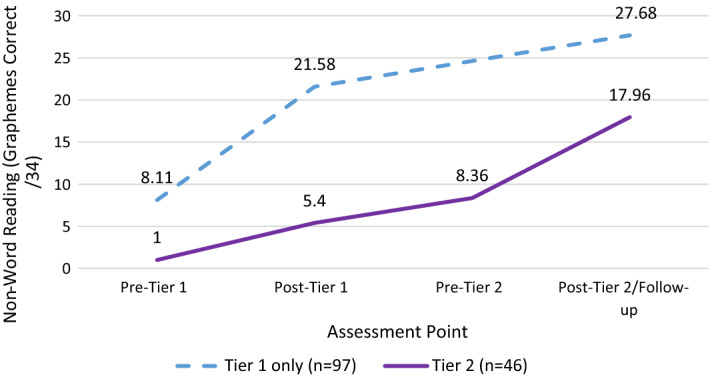
Non-word reading scores over time for Tier 1-only and Tier 2 children

Finally, we compared post-Tier 2 data to the follow-up data for children who participated only in Tier 1 and were not identified for Tier 2 support to determine whether Tier 2 additional support was able to bring children’s skills up to similar levels to their peers who did not require Tier 2. For this analysis, we used data only from the 2019 intake of Group A children (n = 46 Tier 2; n = 97 Tier 1 only), since these children had been in the study for the longest period. In this group of children, “Tier 1 only” children were assessed pre- and post-Tier 1 and then had a follow-up assessment approximately 12 months after their baseline assessment. Tier 2 children were assessed pre- and post-Tier 1 and pre- and post-Tier 2. Figure 5 shows the progression of non-word reading scores over the one-year period. The results show that the implementation of Tier 2 support served to reduce the disparities between groups over time; however, the groups still differed significantly in their reading scores post-Tier 2 (*F*(1141) = 58.90, *p* < 0.001).

**Fig. 6:**
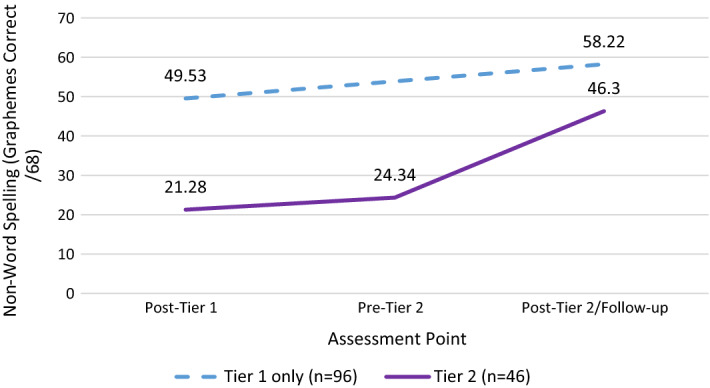
Non-word spelling scores over time for Tier 1-only and Tier 2 children

Figure 6 shows a similar graph to the above but for non-word spelling scores over the one-year period. Note that spelling was not assessed pre-Tier 1. In this case, we see a pronounced impact of Tier 2 in terms of reducing the gap between children in Tier 1 only and those who required Tier 2 support; however, the difference between groups remained significant (*F*(1141) = 39.51, *p* < 0.001).

## Discussion

Understanding effective classroom practices that advance early reading and writing success for children most in need is essential if we are to reduce current educational inequities. In this study, we sought to expand upon earlier pilot trial findings by examining the effectiveness of the Better Start Literacy Approach (BSLA) in a diverse range of school communities. Furthermore, the impact of the age of children in the cohort (also a proxy for the number of months children had spent at school prior to the implementation of the BSLA) on the effectiveness of the approach was investigated. Finally, the study investigated the benefits of implementing Tier 2 small group teaching following 10 weeks of Tier 1 teaching for 5-year-old children who needed more support in acquiring phonological awareness and word decoding skills.

New entrants and Year 1 teachers from 14 schools participated in the study. Schools were randomly chosen for their teachers to receive professional learning and development to implement the BSLA first or to continue with their regular literacy curriculum and then implement BSLA after a period of approximately 10 weeks. Data from 402 5-year-old children who had returned parental research consent forms from these teachers’ classes were analysed. The results demonstrated that after only 10 weeks of teachers implementing the BSLA Tier 1, the children in these classes performed significantly better in phonological awareness, non-word reading, and non-word spelling tasks than their peers whose teachers continued with their usual literacy teaching (which included other types of phonic and phonological awareness programmes). The replication of positive findings from the earlier pilot study with a new cohort of teachers and children is very encouraging. It suggests the BSLA may be an effective early literacy approach across a diverse range of school communities in New Zealand.

Within-group data inspections showed that the BSLA quickly reduced the wide variability in children’s phonological awareness that was evident at school entry through advancing the skills of children with lower-level abilities. Approaches that rapidly accelerate the cognitive skills that support later reading accuracy and comprehension for children who enter school with lower-level skills is particularly important within the New Zealand educational context. The Progress in International Reading and Literacy Study (PIRLS) data consistently show that New Zealand 10- to 11-year-old children demonstrate wide variability in their reading comprehension scores (Mullis et al., 2017). Indeed, the variance between high and low performing readers in New Zealand is one of the largest variances in the OECD countries who participated in this international study*.* Ensuring that early literacy instruction can rapidly advance children’s foundational reading skills regardless of children’s skill level at school entry is a first step towards reducing variability in later reading outcomes.

Importantly, the results suggested that in response to the BSLA teaching, children were learning to use their enhanced phonic and phonological awareness knowledge in decoding and encoding written words, as evidenced by their significantly improved non-word reading and spelling performance. Recently, Wilson et al. (2021) also reported positive outcomes from explicit teaching via a digital game format that focused children’s attention on word decoding and encoding skills. In their study, they observed children’s responses to a digital game intervention that they played for 10–15 min daily for 12 weeks. The game adapted the pace of instruction to the children’s responses for learning groups of phoneme–grapheme matches and the ability to use this knowledge to decode and encode words at the onset-rime level. Participants were 6- and 7-year-old children in the United Kingdom who showed low phoneme awareness skills at the end of their first year at school. Interestingly, detailed analysis revealed that the boys made faster progress through the digital games than girls and therefore engaged in more decoding and encoding practice. A common teaching element between the current study and Wilson’s study is the explicit teaching approach to helping children understand the connection between graphemes and phonemes and then to use this knowledge to decode and encode words. Both approaches provided the children with sufficient practice and feedback to transfer their developing phonic and phonological awareness skills to the reading and spelling process.

Wilson et al.’s study (2021) also demonstrated that children’s phoneme awareness ability at the end of their first year of school was a strong predictor of how the children responded to literacy instruction in Year 2. The current study provides evidence that, with appropriate support and the use of research-informed teaching strategies, class teachers can rapidly advance the phonological awareness and word decoding and encoding skills of the majority of children in their class during their first year at school. In line with Wilson’s findings, this would suggest that advancing these foundational skills from school entry will facilitate children’s continued success with reading and writing in Year 2.

The second question this study addressed was whether the effectiveness of the BSLA differed based on the children’s age within year group at implementation. Following the BSLA, younger children within year groups scored higher than their older class peers in the letter–sound knowledge task. Further, the teaching effect for phoneme awareness in Group A (compared to Group B) was significant for the younger age group alone. There were no age differences in teaching response in the other measures. This stronger response to phonic and phoneme awareness BSLA teaching for younger children in the class contrasts with previous research that shows that older children within a grade level perform better on phonological awareness tasks (Norbury et al., 2016). Younger children in the class achieving higher scores on the letter sound tasks that their older peers in the class was also surprising given the increased exposure the older children had to letter sound and phoneme awareness programmes prior to the BSLA being implemented (Cornelissen & Dustmann, 2019).

The relatively stronger teaching response for younger children cannot simply be explained by the older children reaching a ceiling on the assessment tasks. The finding may be partially explained by the children’s general language ability. Further analysis of CELF core language scores showed that younger children scored significantly higher than older children. However, analysis showing that the advantage for younger children was primarily driven by the performance of younger children in Group A who received BSLA first (rather than young children overall) suggests that general language functioning does not fully explain the result. Rather, there may have been some advantage for children who received the BSLA at the outset of their literacy instruction as opposed to switching from one literacy teaching approach to another. Due to the nature of the research design and the school starting age in New Zealand, younger children in Group A immediately received the BSLA upon school entry. In contrast, older children in their first year at school in Group A and all children in Group B received other types of phonic and phonological awareness programmes prior to BSLA. This shift in approach may have meant that these children had to ‘unlearn’ certain teaching strategies (such as assignment of a character to a letter name) or refine an already established imprecise phonological representation of a given grapheme. The results highlight the importance of the implementation of evidence-based explicit phonic and phonological awareness teaching right from the outset of formal literacy instruction.

A further question this study addressed was understanding the additional benefits from implementing Tier 2 teaching support for 5-year-old children in their first year at school. The results show that Tier 2 resulted in significantly accelerated growth in non-word reading and spelling for children who participated in Tier 2 as compared to a control group. Children in the control group continued to receive the Tier 1 BSLA, and this appeared sufficient to advance their phoneme awareness skills to a similar extent as the children receiving additional Tier 2 small group sessions. Tier 2 small group work showed the greatest benefit in supporting children with their use of enhanced phoneme awareness knowledge in the reading and spelling process. This was particularly evident when examining the reading and spelling growth pattern over a 12-month period of children who received Tier 2 support compared to their peers who had higher level skills at school entry and did not require Tier 2 support. Accelerated growth for children in Tier 2 towards the levels of their peers was clearly evident. This finding is consistent with previous findings (e.g., Carson et al., 2013; Gillon et al., 2020) that some children require greater support to integrate knowledge about a word’s sound structure into their reading and spelling attempts. However, as children were not randomly assigned to Tier 2 vs. control groups in this study, no conclusions around causal relationships can be drawn. The non-random assignment was due to project time constraints and teachers’ desire for as many children as possible to receive Tier 2 support if this was recommended. Further research using a randomised Tier 2 design would provide additional, causal evidence beyond the current results.

The Tier 2 support implemented in the current study built upon and aligned with the Tier 1 teaching. Neitzel et al. (2021) reviewed 65 robust research evaluations of differing interventions (n = 51) aimed at improving reading outcomes for younger struggling readers (K–Year 5). The researchers concluded that significant positive impacts on reading are evident from programmes aligned with a response to intervention framework. In particular, approaches that co-ordinate efforts around evidence-based Tier 1 class teaching with proven tutoring approaches at Tier 2 and Tier 3 show the most promise. Interestingly, Neitzel et al.’s research synthesis demonstrated that teaching approaches that involved trained teacher assistants (bachelor’s degree qualified) implementing small group or individual sessions resulted in similar positive outcomes for struggling readers compared with when qualified teachers delivered the Tier 2 teaching. In the current study, literacy specialists or SLPs delivered the Tier 2 instruction. The effect sizes reported in our study are large in comparison to Neitzel’s studies. The average effect size for the 14 studies Neitzel reviewed (where Tier 2 tutoring replaced their regular small group reading instruction as in the current study) was 0.29. This compares to effect sizes greater than 0.6 for non-word reading and spelling after 10 weeks of Tier 2 teaching in our study. Future research controlling for these types of variables may lead to better understanding of the longer-term cost benefits of rapid acceleration in children’s learning through specialists implementing Tier 2 versus steady growth with well-trained assistants implementing Tier 2.

Change in teacher knowledge was not specifically evaluated in this study. However, the accelerated improvement in children’s skills in response to the BSLA Tier 1 teaching suggest that the robust teacher PLD provided and leadership support for class teachers to enhance their current practice or adopt new strategies contributed to improved learner outcomes. This is consistent with recent findings that a school community approach may well be needed to ensure sustained enhancement or change in teachers’ early literacy teaching practices (Goldfeld et al., 2021). Visible school leadership support, quality teacher professional learning and development, and ongoing coaching may all be necessary.

The findings from this study are very promising. The data suggest that with appropriate support and resources, class teachers can rapidly advance the foundational literacy skills that are critical for early reading and writing success in a relatively short period (10 weeks) for most children. The wide variability in 5-year-old children’s skills evident at school entry can be dramatically reduced through accelerating learning for those with lower-level skills. In addition, quality Tier 2 support following 10 weeks of Tier 1 teaching can rapidly improve outcomes for children with lower oral language ability. Ensuring successful early reading experiences for all children from their first year at school will help these children to reach their learning potential.

## Appendix 1

### Phonological awareness task items

#### Initial phoneme identity

**Table:**

Item number	Item	Target
Practice 1	Duck	/d/
Practice 2	Door	/d/
Test 1	Milk	/m/
Test 2	Mat	/m/
Test 3	Sun	/s/
Test 4	Saw	/s/
Test 5	Kite	/k/
Test 6	Comb	/k/
Test 7	Ball	/b/
Test 8	Boat	/b/
Test 9	Foot	/f/
Test 10	Fire	/f/

#### Phoneme blending

**Table:**

Item number	Item	Target
Practice 1	Cake	CVC
Practice 2	Stop	CCVC
Item 1	Cat	CVC
Item 2	Seat	CVC
Item 3	Mouse	CVC
Item 4	Bun	CVC
Test 5	Flag	CCVC
Test 6	Crab	CCVC
Test 7	Snake	CCVC
Test 8	Train	CCVC
Test 9	Pond	CVCC
Test 10	Bank	CVCC
Test 11	Lamp	CVCC
Test 12	Cast	CVCC

#### Phoneme segmentation

**Table:**

Item number	Item	Target
Demo	Sun	3
Practice 1	Dog	3
Test 1	Tooth	3
Test 2	Cup	3
Test 3	Soap	3
Test 4	Saw	2
Test 5	Flush	4
Test 6	Crab	4
Test 7	Sew	2
Test 8	Star	3
Test 9	Bank	4
Test 10	Lock	3
Test 11	Jump	4
Test 12	Pond	4

#### Letter–sound knowledge

**Table:**

Practice 1	r
Practice 2	j
Test 1	m
Test 2	s
Test 3	k
Test 4	b
Test 5	n
Test 6	f
Test 7	d
Test 8	h
Test 9	p
Test 10	t
Test 11	w
Test 12	g
Test 13	c
Test 14	z
Test 15	l
Test 16	q
Test 17	v
Test 18	y

## Footnotes

1 Funding as part of this project did not cover Tier 2 for the second cohort of children; however, teachers were provided with assistance and teaching resources at the close of the project to implement Tier 2 for these children.
